# Ternary Electrical Memory Devices Based on Polycarbazole: SnO_2_ Nanoparticles Composite Material

**DOI:** 10.3390/polym14071494

**Published:** 2022-04-06

**Authors:** Yingna Zhang, Feng Dou, Yijia Zhou, Xiaofeng Zhao, Jiangshan Chen, Cheng Wang, Shuhong Wang

**Affiliations:** 1 School of Chemical Engineering and Materials, Heilongjiang University, Harbin 150080, China; zhangyingna1997@163.com (Y.Z.); df15196081421@163.com (F.D.); zhouyijia1998@163.com (Y.Z.); 2 School of Electronic Engineering, Heilongjiang University, Harbin 150080, China; zxf80310@126.com; 3 Institute of Polymer Optoelectronic Materials and Devices, State Key Laboratory of Luminescent Materials and Devices, Guangdong Provincial Key Laboratory of Luminescence from Molecular Aggregates, South China University of Technology, Guangzhou 510640, China; msjschen@scut.edu.cn; 4 Key Laboratory of Functional Inorganic Material Chemistry, Ministry of Education, Heilongjiang University, Harbin 150080, China

**Keywords:** ternary memory device, tin dioxide nanoparticle, switching current ratio

## Abstract

In this paper, a D–A polymer (PIB) containing carbazole as the donor group in the main chain and benzimidazole benzisoindolinone as the acceptor group was synthesized by Suzuki reaction. The Suzuki reaction, also known as the Suzuki coupling reaction, is a relatively new organic coupling reaction in which aryl or alkenyl boronic acids or boronic acid esters react with chlorine, bromine, iodoaromatic hydrocarbons or alkenes under the catalysis of zerovalent palladium complexes cross-coupling. A series of devices were fabricated by a spin-coating approach, and the devices all exhibited ternary resistance switching storage behavior. Among them, the composite device with the mass fraction of SnO_2_ NPs of 5 wt% has the best storage performance, with a threshold voltage of −0.4 V and a switching current ratio of 1:10^1.5^:10^4.5^. At the same time, the current of the device remained stable after a 3-h test. Furthermore, after 10^3^ cycles, the current has no obvious attenuation. The device has good stability and continuity. Moreover, the conduction mechanism is further revealed. Inorganic nanoparticle composite devices have splendid memory performances and exhibit underlying application significance in storing data.

## 1. Introduction

The device size of traditional non-volatile memory has reached technical and physical limits. In order to solve this problem, researchers have proposed some new types of nonvolatile memories [1,2,3,4]. Among them, resistive random-access memory (RRAM) has aroused extensive interest due to its simple structure, low power consumption, high performance, fast switching speed, long retention time, and compatibility with semiconductor processes [1,2,3,4]. In recent years, small organic molecules and conjugated polymers have been widely used in non-volatile memory devices due to their simple structure, good processing performance, small size, multi-dimensional storage capabilities, and molecular design [1,2,3,4]. However, polymer memory and traditional silicon-based memory store charges in a different way. That is, under the same applied voltage, the device can respond to a high resistance state (HRS) and low resistance state (LRS), where the HRS and LRS are equivalent to the 0 state and the 1 state in silicon-based devices. This cannot satisfy the high-efficiency storage of rapidly increasing data [5,6,7,8].

In recent years, to ameliorate the information storage capability, RRAM has achieved ternary electrical storage characteristics by selecting active layer materials. The storage capacity can be increased to 3^n^, and the data storage capacity of the device has been significantly improved. Among them, the most widely used active layer material is the donor–acceptor (D–A) polymer. The D–A polymer has become a perfect material for developing novel organic resistive memory equipment due to its structural adjustability, special photoelectrical properties, and the possibility of gradually inducing the charge transfer process [9,10,11,12].

Apart from electrical memory equipment based on pure polymeric materials as the active layer, organic/inorganic nanocomposite materials have been broadly researched by scholars as well. In the current research, most of the composite memory devices show good performance. It not only maintains the splendid properties of polymeric electrical storage devices, but also has the special electrical performances of non-organic nanoparticles [13]. These include, for example, poly(vinyl carbazole):titania quantum dots [14], poly-carbazole-decorated TiO_2_ nanohybrids material [15], polycarbazole:titanium dioxide nanoparticles [16], polymethylmethacrylate:tin oxide nanoparticles [17], and polycarbazole:tin oxide nanoparticles [18]. Among various types of nanoparticles, SnO_2_ NPs is a typical wide bandgap n-type metal oxide semiconductor material, with a bandgap of 3.6 eV and an excitonic binding energy of 130 meV, and good electrical conductivity. SnO_2_ NPs is a semiconductor material with rutile crystal structure. Each Sn atom is located at the center of an approximate octahedron composed of 6 oxygen atoms, forming a stable structure, making it chemically stable. SnO_2_ NPs have a naturally formed bandgap, and many vacancy defects offer a potential way for charge trapping as well, making them candidates for charge–discharge nodes in memory devices. When SnO_2_ NPs are embedded in the polymeric material as the active tier, they can be a carrier trap to improve the resistance switching performance [19,20].

In this paper, we synthesize a D–A type polymer containing carbazole as the donor group in the main chain and benzimidazole benzisoindolinone as the acceptor group through the Suzuki reaction. The prepared memory device shows unique ternary electricity storage behavior. We also prepared different concentrations of the polymer SnO_2_ NPs solution and prepared various energy-storing equipment. The performance test results show that the storage properties of embedded SnO_2_ NPs equipment have been significantly improved, and the electrical bistable switching behavior of the devices can be adjusted by changing the content of SnO_2_ NPs.

## 2. Materials and Methods

### 2.1. Materials

3,6-Dibromo-1,2-phenylenediamine and 2,3-naphthalic anhydride were provided by Beijing Huanling Technology Company. The rest of the reactants and catalyzers (Aladdin Biochemical Technology, & Sinopharm Group Co. Ltd., Shanghai, China) were utilized as they were. We use toluene after removing its water. The rest of the reagents and solvents met experimental requirements (Sinopharm Group Co. Ltd., Shanghai, China) and were utilized as they were and not purified further. SnO_2_ NPs are preserved in a dry N_2_ milieu at a particulate size of 40 nm.

### 2.2. Synthesis of Monomer

**Scheme 1:**
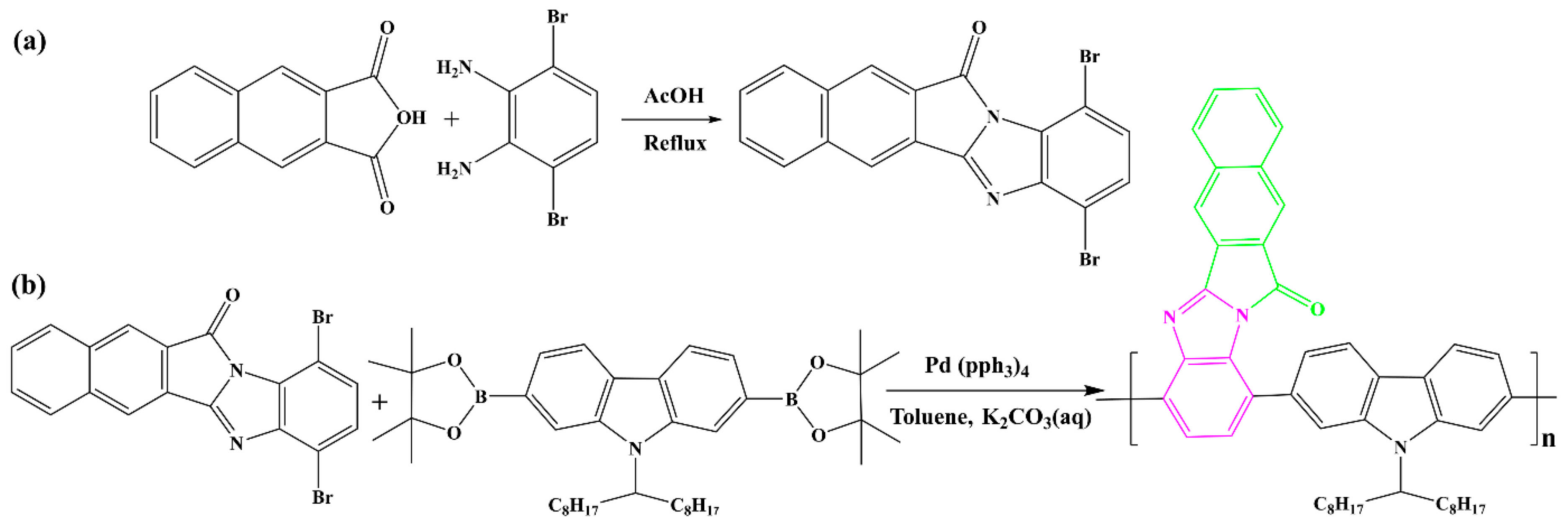
Synthetic route of monomer (**a**) and polymer (**b**).

Scheme 1a presents the synthetic process of 1,4-dibromo-12H-benzo [5,6] isoindolo[2, 1-a] benzimidazole-12-one. Add 3,6-dibromo-1,2-phenylenediamine (0.128 g, 0.5 mmol) and 2,3-naphthalenedicarboxylic anhydride (0.088 g, 0.5 mmol) into a 50-mL Schlenk bottle separately, and we supplemented 15 mL of glacial acetic acid. The solution was subjected to heating and agitation under a constant temperature of 110 °C under the protection of N_2_ for 10 h. Posterior to the reactive process, the blended liquor was transferred into deionized water, and the precipitated crude product was harvested by vacuum filtration. The solution was cleaned by deionization several times and desiccated in a vacuum oven for 12 h. Eventually, the raw product was subjected to purification via silica gel column chromatographic method (petroleum ether, dichloromethane) to acquire a yellow solid (0.64 g). The yield was 64%. Figures S1 and S2 show the NMR spectrum of the monomer.

### 2.3. Synthesis of Polymer

Scheme 1b presents poly[2,7-9-(heptadecan-9-yl)-9H-carbazole-alt-12H-benzo [5,6] isoindolo[2,1-a]benzimidazole-12-one] (PIB). Add the monomer (0.13 g, 0.3 mmol), 9-(1-octylnonyl) carbazole-2,7-bis (pinacol borate) (0.20 g, 0.3 mmol), tetra (triphenylphosphine) palladium [PD(PPh_3_)_4_] (6.90 mg, 0.007 mmol), K_2_CO_3_ (0.27 g dissolved in 1 mL deionized water), and 6 mL toluene were successively added to the Schlenk bottle. The mixed solution was subjected to agitation and heating in N_2_ at 110 °C for 48 h. Subsequently, the liquor was extracted in a repetitive manner using deionized water. It was supplemented with 200 mL of icy methyl alcohol, and the sediment was harvested via a filtering process. Eventually, the product was subjected to purification via Soxhlet extraction with acetone for 48 h and desiccated in a vacuum oven. A grayish-black solid (0.11 g) was obtained. The yield was 51%. Figure S3 shows the ^1^H NMR of the polymer.

### 2.4. Memory Device Preparation

The ITO glass used for storage devices was washed in deionized water, acetone, and methanol in a supersonic bath for 0.5 h. The 3 mg mL^−1^ polymer toluene liquor was prepared. The preparation process of PIB: SnO_2_ NPs composites material was as follows: SnO_2_ NPs were dissolved in N-methylpyrrolidone, and ultrasound for 60 min to prepare dispersions of SnO_2_ NPs with different concentrations. After that, the SnO_2_ NPs dispersion was blended with the pre-treated polymer liquor and sonicated for 60 min. Table 1 presents the diverse proportions of the PIB: SnO_2_ NPs composite solution. Then, the mixed liquor was spin-coated on the ITO glass, rotated at 1200 rpm for 20 s, and then rotated at 3330 rpm for 50 s. The obtained semi-finished product was desiccated in a vacuum oven. The specimen surface was covered with a copper sheet (aperture size of 2 mm), and aluminum was vapor-deposited on the active layer surface under vacuum evaporation conditions (2.0 × 10^−3^ Pa).

## 3. Results and Discussion

### 3.1. Polymer Characterization

**Figure 1:**
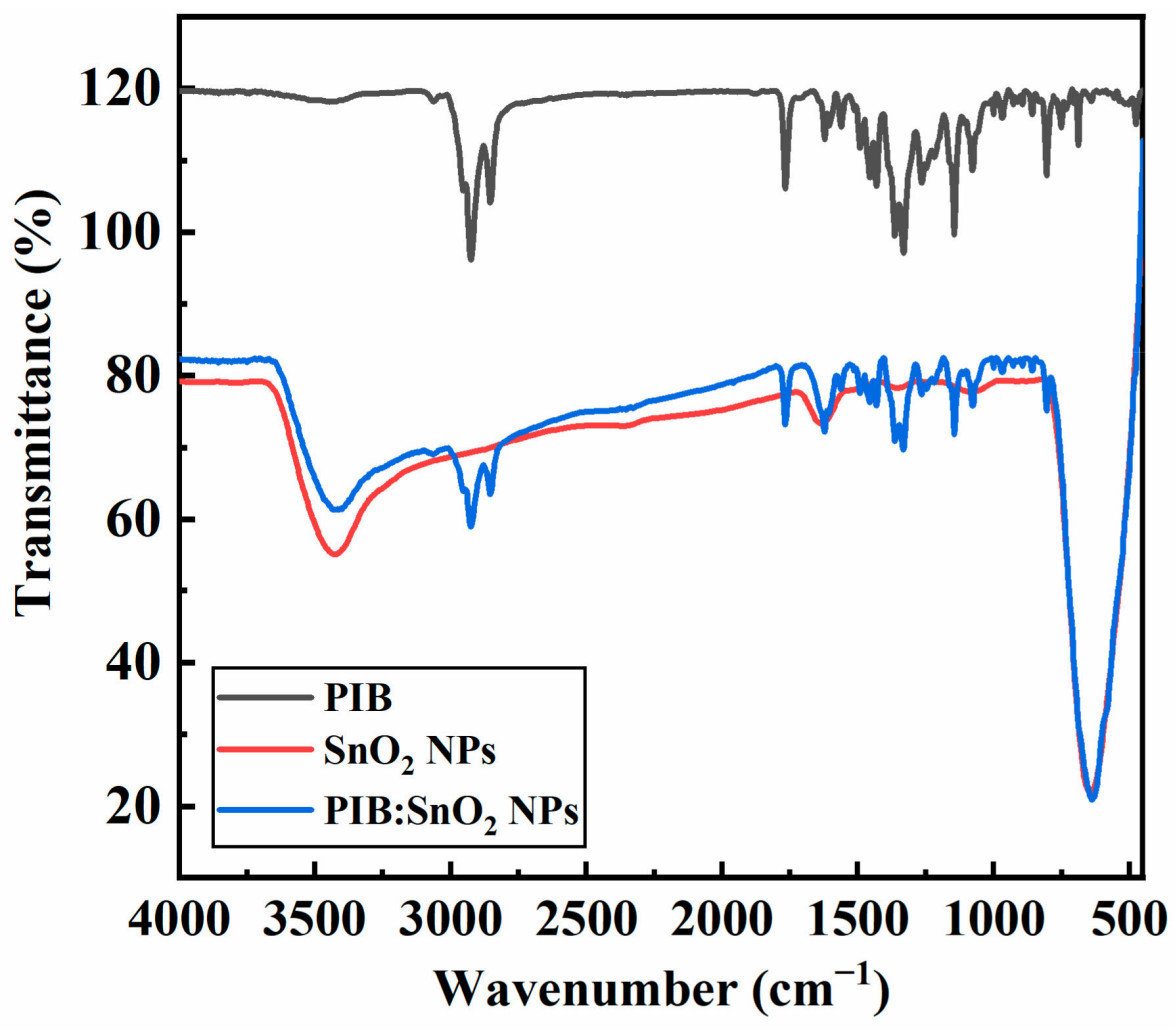
Infrared spectra of PIB and PIB: SnO_2_ NPs.

The infrared spectrum of the polymer is presented by Figure 1. The aromatic C-H stretch vibration absorptive peak (SVAP) is at the wave number 2925 cm^−1^, the aliphatic C-H SVAP is at 2850 cm^−1^, and the wave number 1767 cm^−1^ is C=O. SVAP, 1616-1483 cm^−1^ is C=C SVAP, and C-N SVAP is 1146 cm^−1^ [21]. For SnO_2_ NPs, we can observe that there is an absorption peak at 3430 cm^−1^, which is caused by the vibration of V_O-H_, and the peak observed at 639 cm^−1^ is caused by the vibration of the O-Sn-O group. The small peak at 1394 cm^−1^ corresponds to the adsorption of water and CO_2_ molecules [22]. For the polymer of composite nanoparticles, the peak at 3429 cm^−l^ is related to the interaction of the NH group of the polymer with the oxygen of SnO_2_. There is a peak at 639 cm^−1^ due to the vibration of the O-Sn-O group. The interaction of the polymer with SnO_2_ nanoparticles affects the conjugation length of the polymer, so there is a small change in the infrared spectrum of the nanocomposite. This confirms the incorporation of SnO_2_ into the polymer chain [18].

### 3.2. Molecular Weight and Thermal Stability of Polymer

The distributional status of the polymer molecule weight was identified via the gel permeation chromatographic method (GPC). The value of the mean molecule weight (${\bar{M}}_{n}$) was 24,450, the weight of the mean molecule weight (${\bar{M}}_{w}$) was 31,545, and its distributional index of the molecule weight was 1.29. To study the thermostability of the polymerization, we used thermogravimetric analysis (TGA) to examine the thermostability of the polymer. The outcomes show that the polymeric material displayed satisfactory thermostability. When the temperature reaches 615 °C, the polymer can maintain over half of the initial weight (as shown in Figure S4).

### 3.3. Optical and Electrochemical Properties

**Figure 2:**
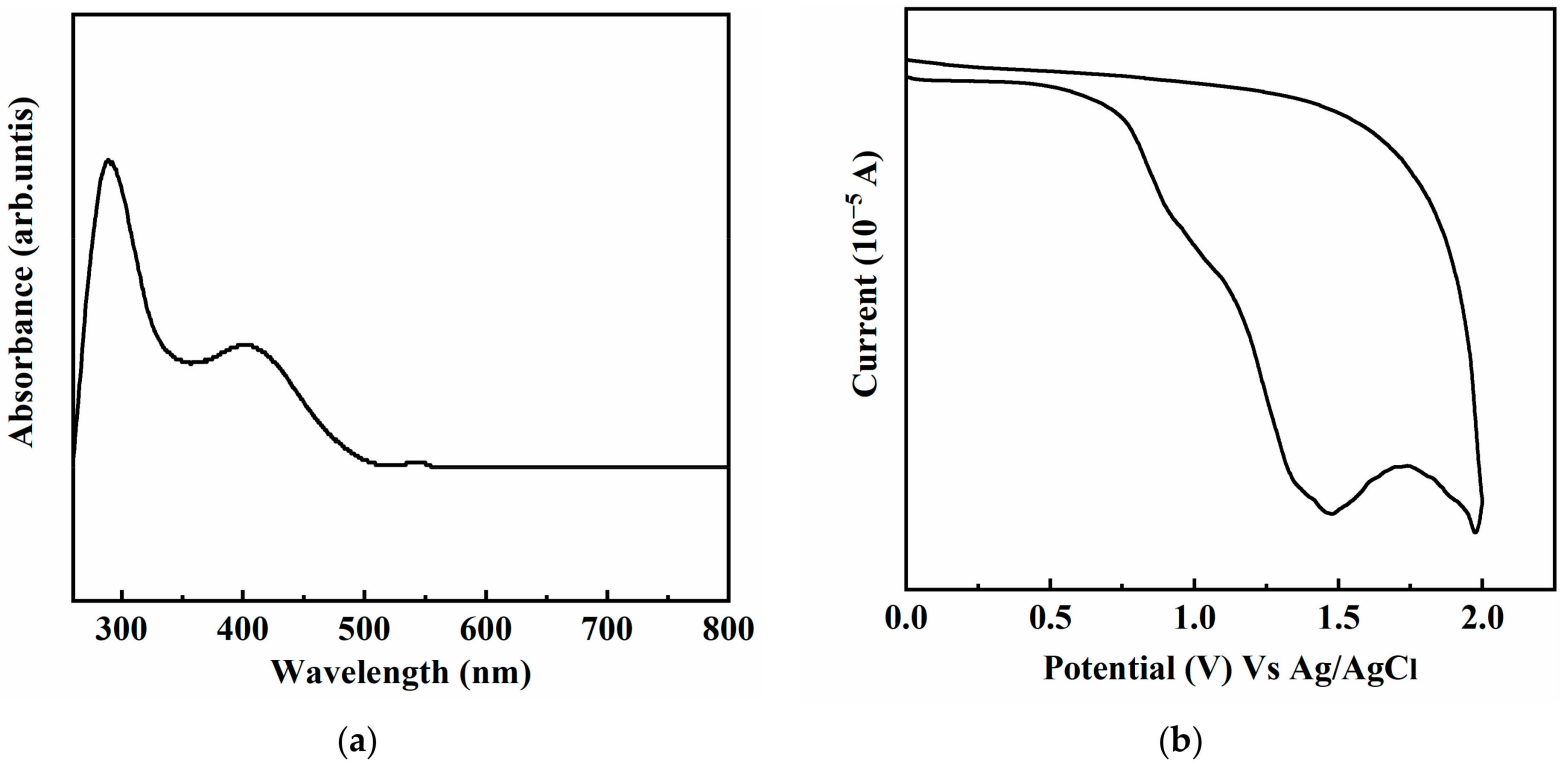
(**a**) UV absorption spectrum of PIB. (**b**) CV curve of PIB.

The absorption spectrum of the polymer in N-methylpyrrolidone (NMP) liquor is shown in Figure 2a. The polymer has a wide absorption band at 260 nm~470 nm, and its maximum absorption peak is at 292 nm. The high-wavelength absorption band is caused by the n-π* transition of the D–A molecule and the inherent charge transfer within the molecule. The absorption edge wavelength of the polymer appears at 488 nm [23]. The optical band gap of the polymeric material was calculated by the formula *E*_g_^opt^ = 1240/*λ*_onset_, (Table S1).

The electrochemical performance of the polymer was identified via CV, as presented by Figure 2b. The energy levels of HOMO and LUMO were computed via the original oxidative potential (E_onset_) and optical band gap (E_g_^opt^). The calculation results are shown in Table 2. The results show that the injection of holes into the HOMO of the polymer by the ITO electrode is more likely to occur than the injection of electrons into the LUMO of the Al electric pole. Therefore, the polymer is a p-type substance, and the conduction process is mainly hole injection. This experimental calculation result is deviated from the calculation result by density functional theory. This may be because the theoretical calculation is the molecular unit structure, whereas the experimental result is the long-chain polymer. Moreover, solvents and electrolytes might influence the outcomes [24].

### 3.4. XRD of SnO_2_ NPs and Micrograph of the Device

**Figure 3:**
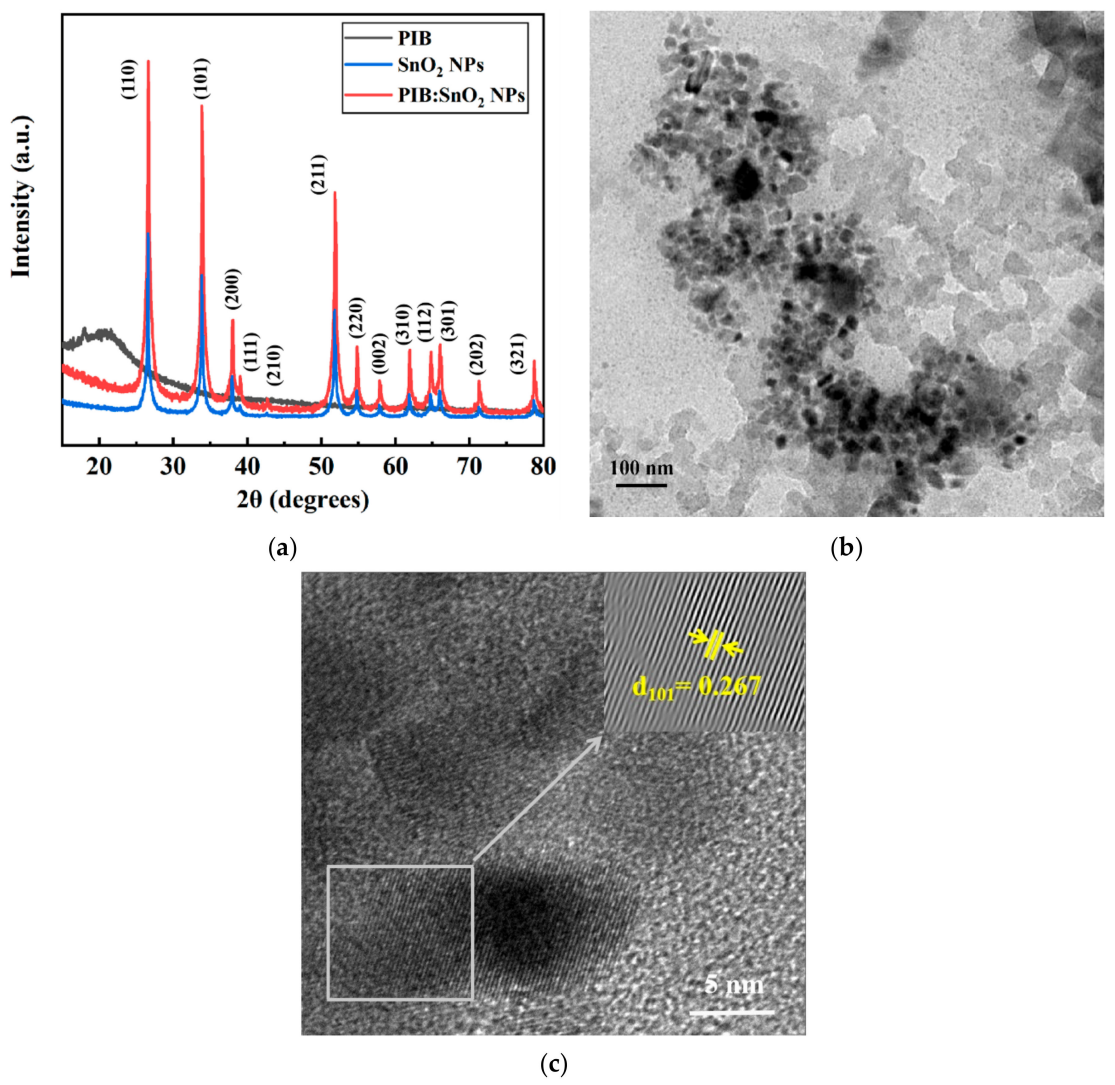
(**a**) XRD spectrum of SnO_2_ NPs and PIB: SnO_2_ NPs. (**b**) TEM of SnO_2_ NPs dispersed in PIB toluene solution. (**c**) High-resolution TEM images.

Figure 3a shows the XRD pattern of SnO_2_ NPs, and its diffraction peaks appear at 26.61°, 33.89°, 37.95°, 38.97°, 42.64°, 51.78°, 54.76°, 57.82°, 61.87°, 64.72°, 65.94°, 71.28° and 78.71°, respectively. The positions of these diffraction peaks are consistent with the international diffractional data standard card JCPDS: No. 41-1445, revealing that SnO_2_ NPs are a tetragonal rutile structure. The above diffraction peaks correspond to the (110), (101), (200), (111), (210), (211), (220), (002), (310), (112), (301), (202), and (321) crystal planes of rutile SnO_2_ NPS, respectively [18,25]. The peaks of pure PIB are at 18° and 21.7°, indicating that the amorphous and polymer chains of the polymer are parallel to each other [22].The XRD peaks of the PIB: SnO_2_ NPs composites indicated the presence of SnO_2_ NPs in the polymer PIB matrix, and the crystallinity of the polymer was improved after the incorporation of SnO_2_ nanoparticles. All peaks of SnO_2_ NPs exist in the composites and no new peaks are generated [22].We calculate the average size of nanoparticles by using the Scheerrer Equation (1) [18]

(1) D=kλ/β cosθ

where *D* is the crystallite size (nm), k is a constant 0.9, λ is the X-ray wavelength, and β is is the half width, and θ is the diffraction angle. The average size of the nanoparticles is shown in Table 2.

As shown in Figure 3b, SnO_2_ NPs are randomly distributed in the polymer toluene solution. Figure 3c is a high-resolution TEM showing lattice fringes with a d-spacing of 0.267 nm, which are the (101) planes of SnO_2_ NPs. This also proves the existence of nanoparticles, the size of which is around 40 nm.

### 3.5. Structure and Cross-Section Possessed by Memory Devices

**Figure 4:**
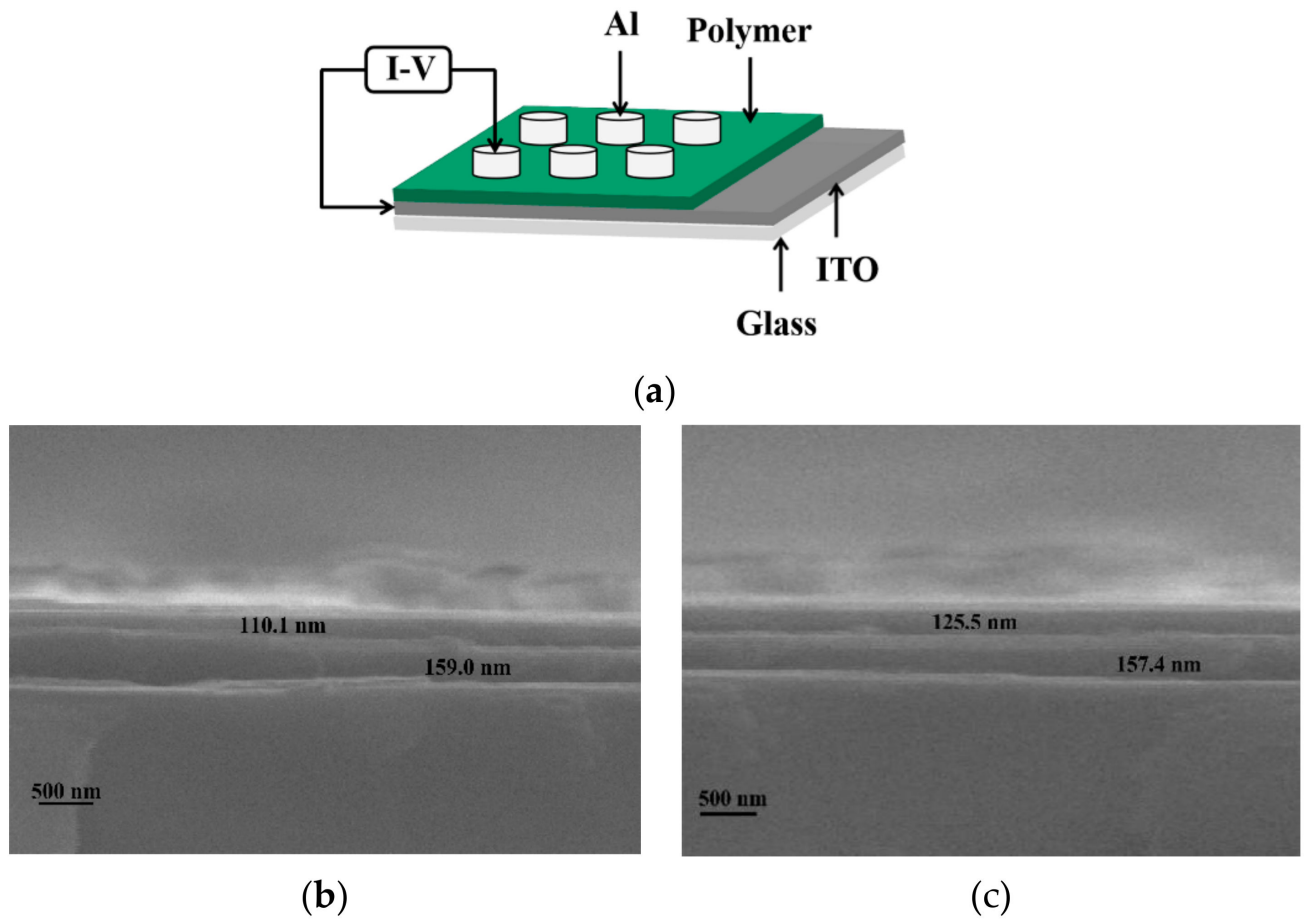
(**a**) The structure of the device. (**b**) The cross-sectional SEM image of the PIB film. (**c**) The cross-sectional SEM image of the PIB: SnO_2_ NPs composite film.

Figure 4a is a structural illustration of the system. Figure 4b,c show the scanning electron microscope (SEM) images before and after polymer composite SnO_2_ NPs, respectively. We can observe the illustration that the sandwich structure is clear, which comprises glass, ITO, and the active layer. The thicknesses of the polymer film and the composite film were 105.3 nm and 110.1 nm, respectively.

### 3.6. Characterization of the Memory Devices

**Figure 5:**
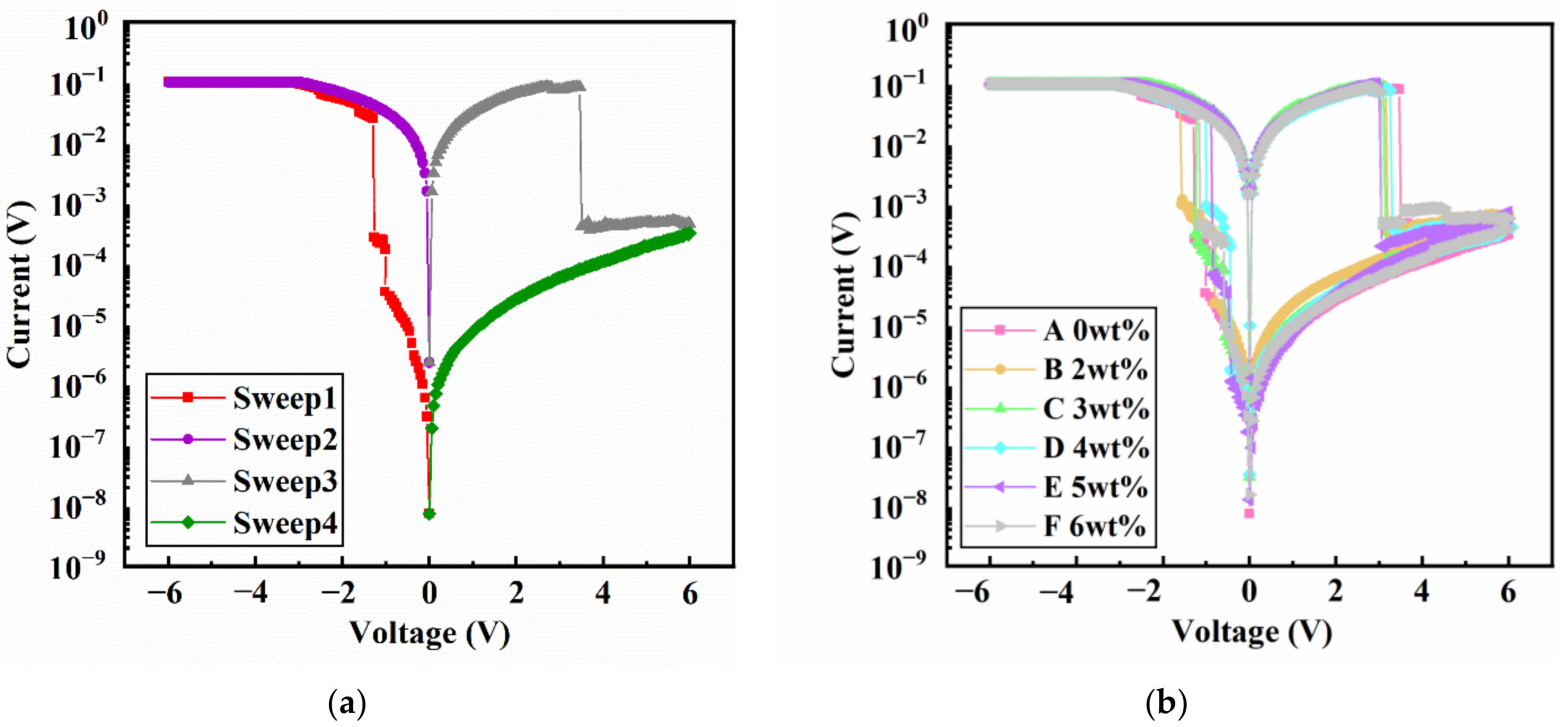
(**a**) I-V curve of ITO/PIB/Al. (**b**) I-V curve of ITO/PIB: SnO_2_ NPs/Al at different SnO_2_ NPs composite concentrations.

For the sake of analyzing the roles of nanoparticle doping levels in polymer memory properties, we tested the properties of the ITO/PIB/Al device (Sample A) before compounding. Figure 5a presents the I-V feature curve of the polymer storage system. When we apply a sweep voltage of 0 to −6 V, the system is initially in a low conduction status (OFF). When the scan voltage is −1.01 V and −1.39 V respectively, the current of the device shows two obvious jumps, corresponding to the (intermediate status) ON1 status and (high conductivity status) ON2 status, as presented by Sweep 1. The device exhibits three different conduction statuses under negative scanning, from the OFF status to the ON1 status and then to the ON2 status. The transformation process of these currents is the “write” process of the storage system. Currently, the switching current ratio is (OFF/ON1/ON2) is 1:10^0.7^:10^3^. Next, as shown in Sweep 2 in the figure, it can still be maintained in the ON2 status under the sweep voltage of −6 to 0 V. This process can be considered as a “read” process, indicating the typical non-volatility of the memory effect [1,2,3,4]. When we apply a reverse sweep voltage of 0 to 6 V, it suddenly drops from the ON2 status to the OFF status (3.5 V), as shown in Sweep 3. This stage is considered to be an “erasing” process. However, under the last scanning voltage of 6 to 0 V, the system is still OFF, which is considered as a “rewrite” process. The re-writable electrical features produced by the four-step scanning constitutes a “write-read-erase-reread” (WRER) cycle process, indicating that the device is a flash-type non-volatile memory [1,2,3,4,26].

For that sake of analyzing the influence of the concentration of SnO_2_ NPs on the memory properties of memory devices, we prepared devices in which the active layer is a mixed solution of PIB: SnO_2_ NPs with diverse amounts. As presented by Figure 5b, it resembles the ITO/PIB/Al devices. Under the scan voltage of 0 to −6 V, the current of all devices also undergoes two transitions, from the OFF status to the ON1 status, and afterwards to the ON2 status. From −6 to 0 V, the current remains in the ON2 state. When a scan voltage of 0 to 6 V is utilized, the system changes from the ON2 status to the OFF status. The OFF/ON1/ON2 current ratios of specimens B, C, D, E, and F were 1:10^1.8^:10^3.2^, 1:10^1.1^:10^3.8^, 1:10^1.5^:10^4.3^, 1:10^1.5^:10^4.5^, and 1:10^1.3^:10^3.4^, respectively. And the liminal values were −0.8 V, −0.6 V, −0.4 V, −0.4 V, and −0.6 V, respectively. Sample E has the lowest liminal value and the highest closing current ratio.

**Figure 6:**
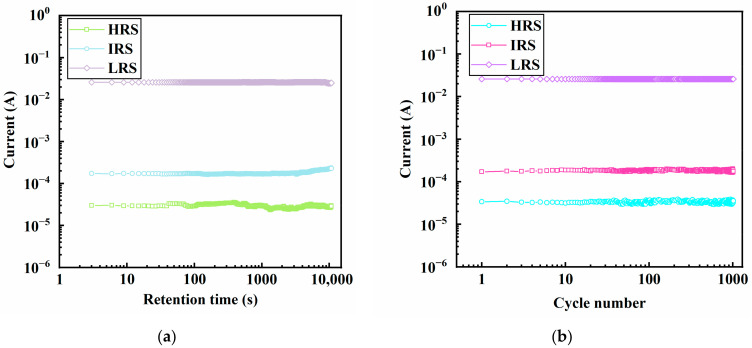

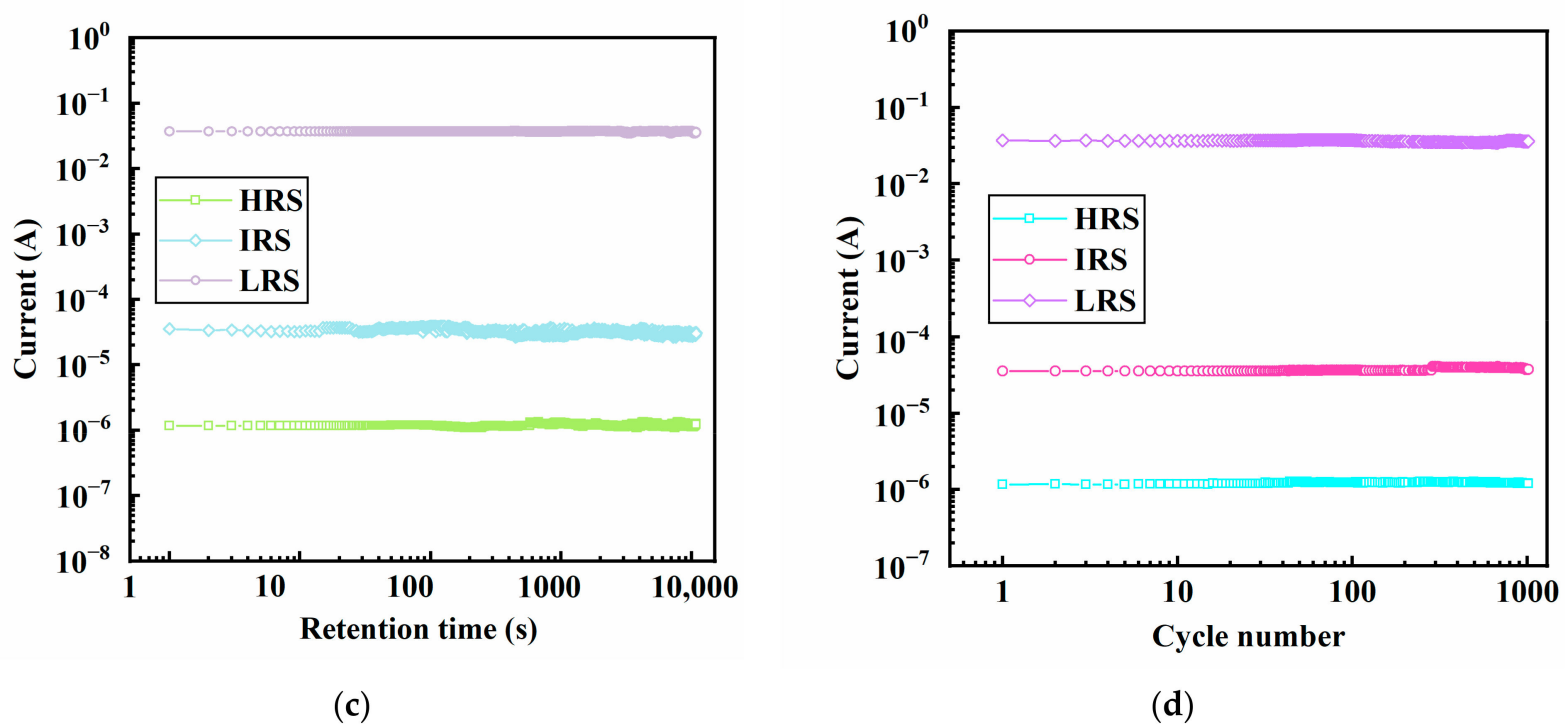
(**a**) The retention performance. (**b**) Endurance performance of the sample A device; (**c**) The retention performance. (**d**) Endurance performance of the sample E-based device.

With an unchanged pressure of −1 V, the currents of the 3 statuses of the device almost changed significantly within 3 h (Figure 6a,c). This shows that the equipment displays satisfactory steadiness. In addition, under the reading pulse of −1 V, after 10^3^ cycles (Figure 6b,d, pulse width is 1 ms, pulse width is 2 ms), the current doesn’t remarkably decay. This shows that the read cycle exerts no influence on the state of HRS, IRS, and LRS, which facilitates the long-term non-volatile data memory. This shows that the memory performance has high durability, and the possibility of misreading errors is very low. In conclusion, ITO/PIB: SnO_2_ NPs/Al storage devices have better system performance. By adding SnO_2_ NPs, the threshold voltage of the device is reduced from −1.01 V to −0.40 V, and the switching current ratio is elevated by 2 orders of magnitude.

### 3.7. Switching Mechanism of Devices

**Figure 7:**
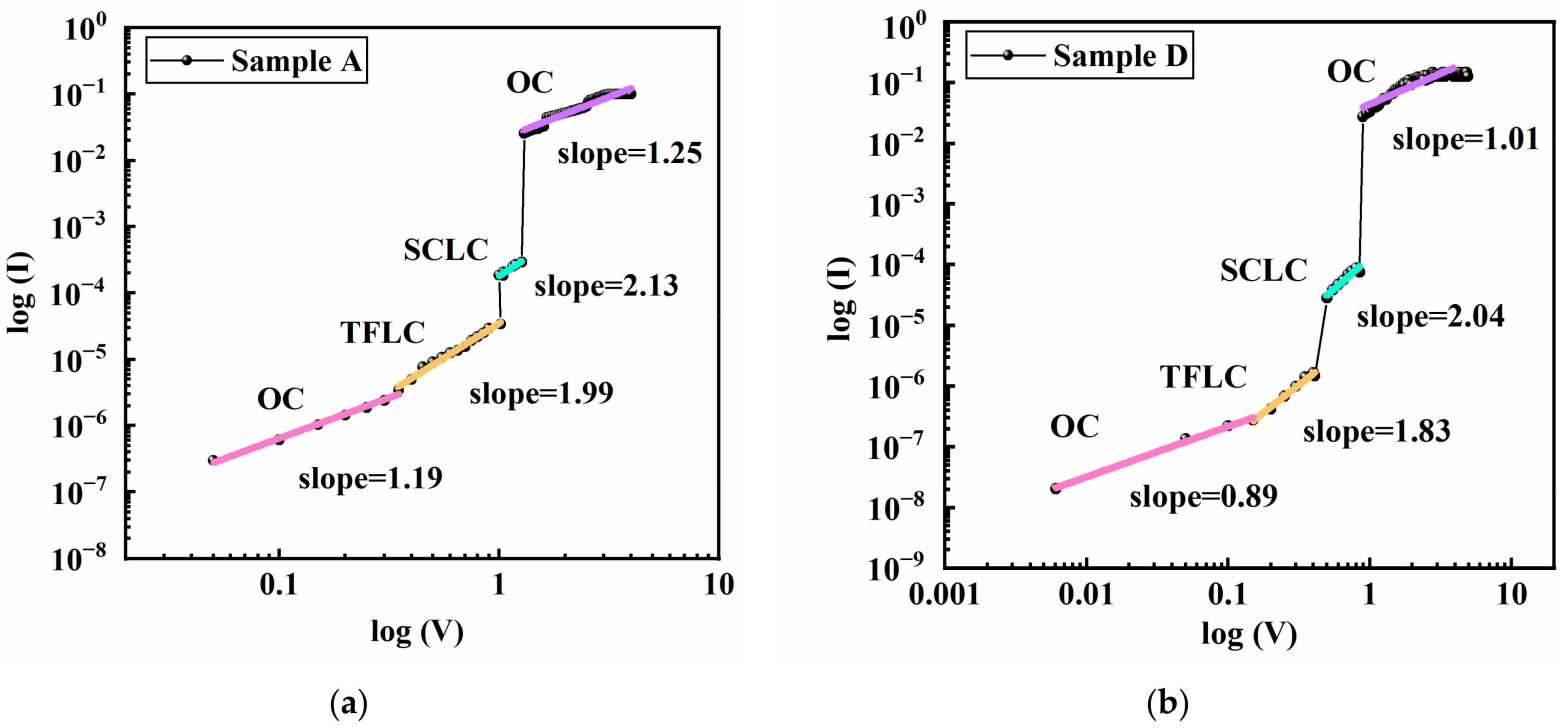
(**a**) The linear fitting curve of the device ITO/PIB/Al. (**b**) The linear fitting curve of the device ITO/PIB: SnO_2_ NPs/Al.

For the sake of exploring the conductance switching process of the two devices, we used the log–log double logarithm to draw the I-V characteristic curve and fitted the corresponding curve fitting (Figure 7). In the low bias voltage area, the current displayed the ohmic conduction (OC) feature, which was produced by the heat excitation of the filled trap. With the gradual voltage elevation, the traps produced by the structure flaws are progressively filled. This area is referred to as the trap filling limited current (TFLC). The injected carriers progressively fill the traps, decrease the bandgap, and elevate the carrier amount. If the voltage elevates to the first liminal value, the whole traps in the film will be filled, and the conductance of the membrane will rapidly jump to a higher level. It is dominated by space charge limited current (SCLC). When the voltage elevates to the second liminal value, carriers cumulate to produce an electroconductivity path. The carriers flow along the path with the lowest electroresistance, hence electroconductivity filaments occur in the charge trapping process [27,28,29].

### 3.8. Device Switching Mechanism

**Figure 8:**
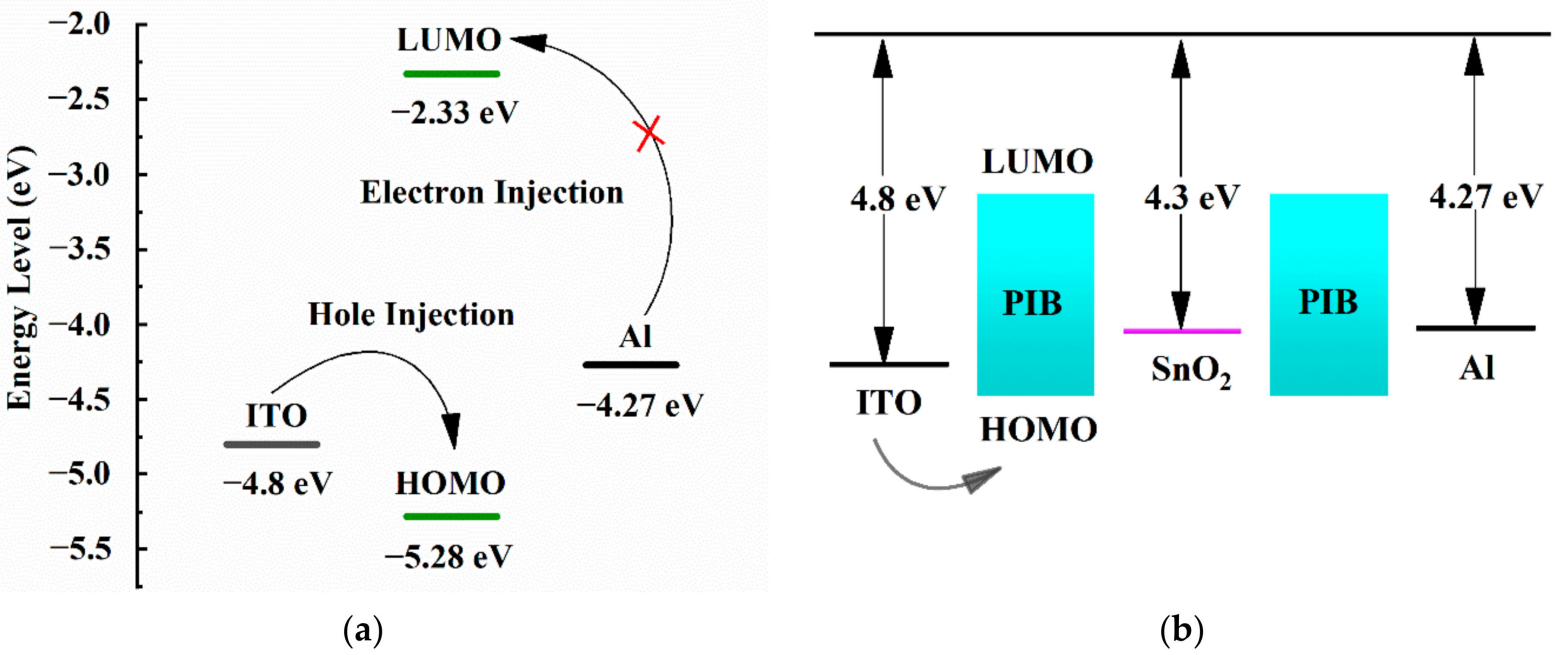
(**a**) Energy level diagram and electron migration process of ITO/PIB/Al. (**b**) Energy level diagram and electron migration process of ITO/PIB: SnO_2_ NPs/Al.

For the sake of better understanding the switching process of the memory system and the corresponding CT process, the density functional theory (DFT) of the B3LYP/6-31G basis set was utilized to calculate the energy level and ESP of the polymeric material [30,31]. The energy barrier between Al electric pole and LUMO energy level is remarkably greater relative to the energy barrier between the ITO electric pole and the HOMO energy level, indicating that the conduction of the equipment is mainly hole injection (Figure 8a). As shown in Table 3, HOMO is primarily in the group that donates electrons, and LUMO is mainly on the electron acceptor. A continuous open channel of positively charged molecules ESP (blue and white) is formed on the entire conjugated main chain, through which carriers can move freely. On the contrary, the negative ESP area (red) generated by the benzimidazole and benzisoindolinone receptors can act as a “charge trap” to prevent the movement of carriers [32]. As the voltage increases, the carriers injected from the electric pole will accumulate in the vacant area between the electric pole and the active tier because of the flaws in the membrane. Therefore, the carriers don’t have enough energy to overcome the potential barrier, and the equipment is in the OFF status. However, as the scan voltage increases (V_thl_), carriers display a donor-to-acceptor movement, filling the trap. Because benzisoindolinone ketone has a more potent electronic absorption capability in contrast to benzimidazole, benzisoindolinone ketone needs to be injected with more energy. The trap quickly generated by benzimidazole is quickly filled, causing the current to realize the switching from the OFF status to the ON1 status. When the voltage is increased to V_th2_, the traps created by the benzisoindolinone are filled, forming a “trap-free” milieu, and then the CT complex is formed. Therefore, the device transitions from the ON1 status to the ON2 status. Overall, the CT complex produced displays metastability. When the electrical field is terminated or reversed, the CT complex will degrade, and the equipment won’t sustain the ON status [33,34].

Given the restricted charge transmission ability of the polymer, posterior to the doping of SnO_2_ NPs in the active tier, SnO_2_ NPs act as electron-trapping centers and are applied to the polymeric active membrane to reinforce charge injection and trapping. As presented by Figure 8b, due to the low work function of SnO_2_ NPs, the Al electric pole and SnO_2_ NPs form an ohm connection at the HOMO level of the polymer. The electron injection of the Al electric pole to the SnO_2_ NPs is better in contrast to the hole injection of ITO. When the voltage reaches a certain value, hole injection occurs, and the injected carriers are captured by SnO_2_ NPs on the surface of the composite film, and a space charge layer is formed. When the liminal value is attained, the carriers overcome the energy barrier and inject into the polymer LUMO energy level along the SnO_2_ NPs. This causes an abrupt current elevation. The content of SnO_2_ NPs in the device will affect the valid distance of neighboring SnO_2_ NPs, promote the trapping behavior of carriers, and make the equipment exhibit different conduction behaviors. Therefore, the ITO/PIB: SnO_2_ NPs/Al device exhibits similar electrical bistable nonvolatile random storage behavior at different SnO_2_ NPs composite concentrations.

## 4. Conclusions

In summary, this study uses spin-coating technology to prepare electrical bistable devices from PIB: SnO_2_ NPs composites. The influence of SnO_2_ NPs concentration on device performance was studied, and different flash-type nonvolatile ternary electrical storage devices were obtained. Compared with ITO/PIB/Al, ITO/PIB: SnO_2_ NPs/Al increases the switching current ratio (from 1:10^0.7^:10^3^ to 1:10^1.5^:10^4.5^) and reduces the threshold voltage (from −1.01 V to −0.4 V), and ensures a low misreading ratio and rapid response. Moreover, the current of the ITO/PIB: SnO_2_ NPs/Al device has no obvious degradation within 3 h. Under the read pulse of −1 V, the current of the device has no obvious attenuation for up to 10^3^ cycles. Therefore, the low liminal value of voltage, high switching current ratio, and splendid steadiness make the PIB: SnO_2_ NPs composite display underlying application full of potential to serve as a dominant form of novel electrical storage equipment.

## Figures and Tables

**Table 1:** Different proportions of PIB: SnO_2_ NPs composite film.

Sample	SnO_2_ (mg)	NMP (mL)	PIB (mg)	Toluene (mL)	SnO_2_ Mass Fraction (wt%)
A	0	0	3	1	0
B	0.061	1	3	1	2
C	0.125	1	3	1	3
D	0.187	1	3	1	4
E	0.261	1	3	1	5
F	0.333	1	3	1	6

**Table 2:** Grain size estimation of SnO_2_ NPs.

Peak (2θ)	FWHM	Crystallite Size (nm)	Average Size (nm)
26.57	0.176	45.9	
33.87	0.208	39.6	40
37.93	0.201	41.4	
51.77	0.260	33.6	

**Table 3:** DFT calculates the molecular ESP and molecular orbital of the basic unit of PIB.

	ESP	HOMO	LUMO
PIB	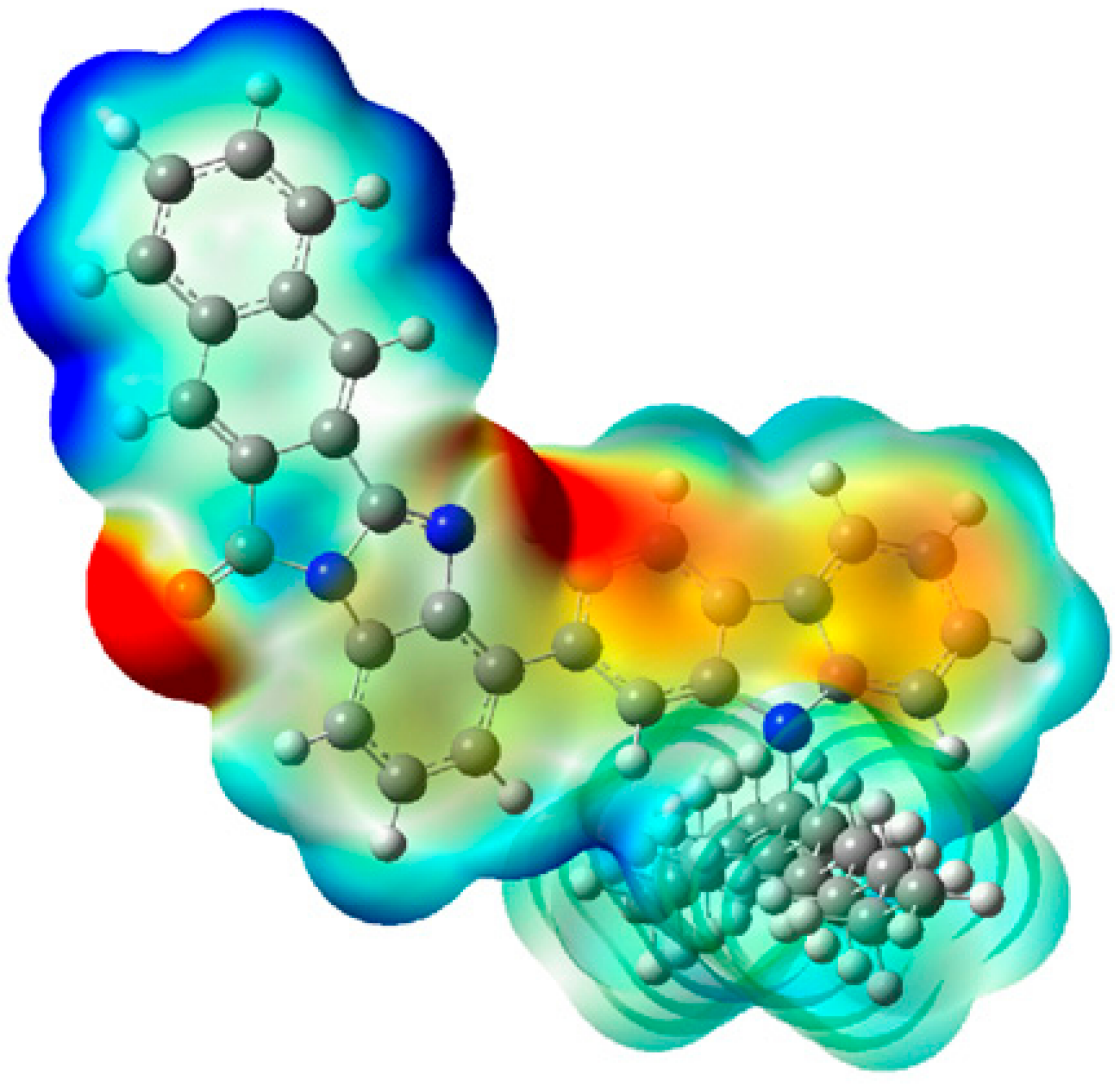	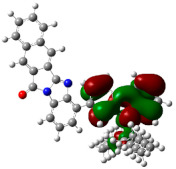	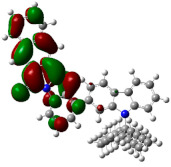

## Data Availability

Data is available upon request.

## Supplementary Materials

The following supporting information can be downloaded at: https://www.mdpi.com/article/10.3390/polym14071494/s1, Figure S1: ^1^H NMR spectrum of monomer; Figure S2: ^13^C NMR spectrum of monomer; Figure S3: ^1^H NMR spectrum of PIB; Figure S4: TGA curves of PIB; Table S1: Optical and electrochemical properties of polymers.
