# Supplementary material for: Ternary Electrical Memory Devices Based on Polycarbazole: SnO2 Nanoparticles Composite Material
Source: Polymers (Basel). 2022 Apr 6;14(7):1494. doi: 10.3390/polym14071494 (PMC9002687; doi:10.3390/polym14071494)
Supplement: Supplementary file 1 [file polymers-14-01494-s001.zip › polymers-1646429-supplementary.pdf]

polymers

MDPI

Supplementary Materials: Ternary electrical memory devices based on polycarbazole:SnO_2 nanoparticles composite material

Yingna Zhang [1], Feng Dou [1], Yijia Zhou [1], Xiaofeng Zhao [2], Jiangshan Chen [3], Cheng Wang [1,4,*] and Shuhong Wang [1,*]

[1] School of Chemical Engineering and Materials, Heilongjiang University, Harbin 150080, P. R. China;
[2] School of Electronic Engineering, Heilongjiang University, Harbin 150080, P. R. China;
[3] Institute of Polymer Optoelectronic Materials and Devices, State Key Laboratory of Luminescent Materials and Devices, Guangdong Provincial Key Laboratory of Luminescence from Molecular Aggregates, South China Uni-versity of Technology, Guangzhou 510640, P.R. China;
[4] Key Laboratory of Functional Inorganic Material Chemistry, Ministry of Education, Heilongjiang University, Harbin 150080, P. R. China; wangc_93@163.com (C.W.)
* Correspondence: wangshuhong@hlju.edu.cn (S.W.); wangc_93@163.com (C.W.)

1. Instrumentation and characterization

By using Bruker advance 400 NMR spectrometry detector and $CDCl_3$ as solvent, 1H NMR and 13C NMR spectra were identified at a resonance frequency of 400 MHz. Fourier transform infrared spectroscopy (FT-IR) was used to identify the polymeric material by Magna-IR560 infrared spectrometer. The spectral range was 4000–450 wavenumbers, and the resolution was 2 cm^{-1}. Thermogravimetric analysis (TGA) was determined via a 10-mg solid powdered specimen under N_2 at a temperature increase rate of 10°C/min (PerkinElmer Pyris 6 TGA). The UV-vis absorptive spectrum was measured with a Shimadzu UV-3600 spectral photometer under ambient temperature. The cyclic voltammetry (CV) measuring was completed via a CHI 660E electrochemistry work station with 0.1 M Bu4NClO4/CH3CN liquor as the electrolytic solution at 50 mV/s. The X-ray diffraction (XRD) measurement is performed with a polycrystalline X-ray diffractometer. The SEM image was captured via the S-4700 SEM under atmospheric conditions, Keithley 4200-SCS was utilized to identify the current-voltage (I-V) features of the storage device.

2. $^{1.}$H NMR and ^{13}C NMR spectra of monomer

1H NMR (400 MHz, CDCl3): δ(ppm)8.45(s, 1H), 8.39(s, 1H), 8.00(t, J = 8.5Hz, 2H), 7.73–7.61(m, 2H), 7.37(s, 2H). 13C NMR (101 MHz, CDCl3): δ(ppm)158.73 (C18), 150.60 (C6), 143.15 (C5), 135.15 (C7), 132.69 (C15), 132.39 (C10), 132.19 (C17), 132.13 (C8), 132.01 (C14), 129.85 (C16), 128.34 (C12), 127.42 (C3), 127.16 (C2), 127.12 (C11), 123.82 (C13), 120.30 (C9), 113.36 (C4), 106.47 (C1).

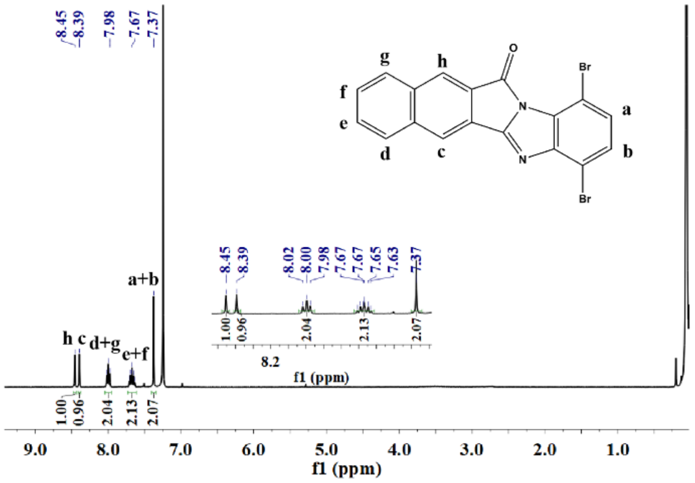

Figure S1. ^{1}H NMR spectrum of monomer.

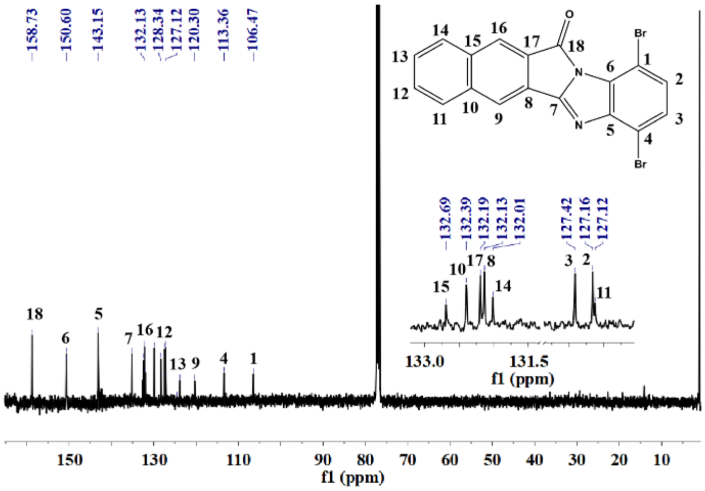

Figure S2. ^{13}C NMR spectrum of monomer.

3. $^{1.}$H NMR spectra of PIB

^{1}H NMR (400 MHz, $CDCl_3$): δ(ppm)9.28–7.49 (m, 15H), 4.68 (dd, J = 122.2, 48.6 Hz, 1H), 1.96 (dd, J = 106.5, 48.8 Hz, 4H), 1.33–0.89 (m, 24H), 0.77 (s, 6H).

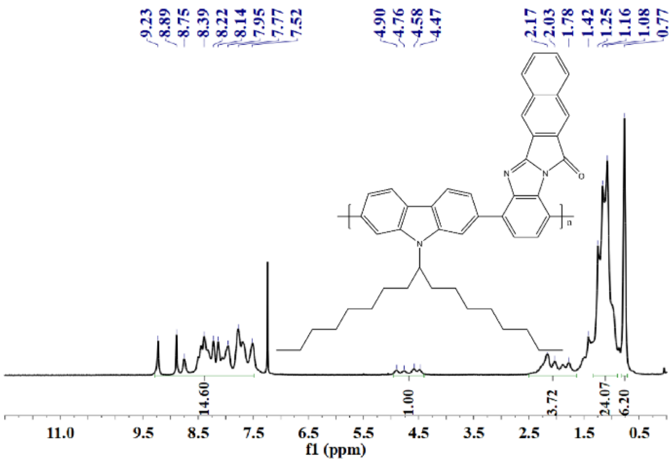

Figure S3. ^{1}H NMR spectrum of PIB.

4. Molecular weight and thermal stability of polymer

The value of the mean molecule weight ($\overline{M_n}$) was 24,450, the weight of the mean molecule weight ($\overline{M_w}$) was 31,545, and its distributional index of the molecule weight was 1.29. The 10 mg specimen was progressively heated to 800°C at a temperature rise speed of 10°C /min in N_2. The outcomes show that the polymeric material displayed satisfactory thermostability. When the weight loss is 10%, the thermal decomposition temperature T_d is higher than 460°C.

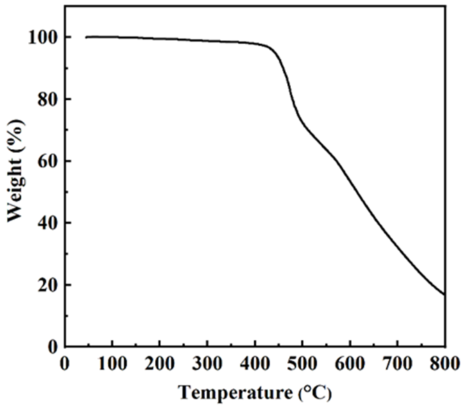

Figure S4. TGA curves of PIB.

5. Optical and electrochemical properties

Table S1. Optical and electrochemical properties of polymers.

	$T_{d,10\%}$(℃)[a]	λ_{onset}(nm)[b]	E_{onset}(V)[c]	E_g^{opt}(eV)[d]	E_{HOMO}(eV)[e]	E_{LUMO}(eV)[e]
PIB	460	488	0.55	2.54	−4.97	−2.43

a $T_{d,10\%}$: Decomposition temperature at 10% thermal weight loss under N_2.
b λ_{onset}: Starting wavelength of polymer solution.
c E_{onset}: The onset potential of the CV curve.
d E_g^{opt}=1240/λ_{onset}.
e E_{HOMO}= −(E_{onset} vs.Ag/AgCl−$E_{ferrocene}$+4.8) eV; E_{LUMO}=E_{HOMO}+E_g^{opt}
